# Das Nest als Umwelt. Eine historische Epistemologie des Nestbauinstinkts in der Schwangerschaft

**DOI:** 10.1007/s00048-020-00285-1

**Published:** 2020-12-01

**Authors:** Lisa Malich

**Affiliations:** Institut für Medizingeschichte und Wissenschaftsforschung, Universität zu Lübeck, Königsstr. 42, 23552 Lübeck, Deutschland

**Keywords:** Geschichte der Reproduktion, Wissensgeschichte, Tier-Studien, Gender, Sorgearbeit, History of reproduction, History of knowledge, Animal studies, Gender, Care work

## Abstract

In heutigen Schwangerschaftsratgebern ist oft von einem Nestbauinstinkt zu lesen. Demnach würden Schwangere von einem Trieb ergriffen, die passende Umwelt für ihr Kind zu gestalten, also Babyausstattung zu kaufen oder die Wohnung zu putzen. Dabei bildet das Konzept des Nestbauinstinkts eine spezifische Wissenskonfiguration: Während es im populären Bereich verbreitet ist, nimmt es im wissenschaftlichen Bereich eine marginale Position ein. Im vorliegenden Beitrag soll der historischen Epistemologie dieser Wissensform nachgegangen werden. Im Vordergrund stehen folgende Fragen: Wie formierte sich das Wissen um einen Nestbauinstinkt in der Schwangerschaft? Auf welche Weise wurde das Nest als spezifische natural-anthropogene Umwelt hergestellt? Und inwiefern interagieren hier Vorstellungen von Geschlecht und Umwelt? Dazu nimmt die wissensgeschichtliche Analyse die Perspektive einer *longue durée* vom 19. Jahrhundert bis heute ein. Die Untersuchung ergibt eine graduelle Feminisierung des Umweltkonzepts im Wissen des Nestbauinstinkts. Während er im 19. Jahrhundert oft als männliches Verhaltensmuster galt und das Nest ein Analogon zum Wohnhaus bildete, transformierte sich der Instinkt in den ersten Jahrzehnten des 20. Jahrhunderts zu einer primär weiblichen Eigenschaft, bei der das Nest für den Innenraum des Zuhauses stand. Dabei zirkulierte das Wissen zwischen verschiedenen Bereichen, wofür eine maßgebliche Bedingung war, dass sich das Nest zum ‚metaphorischen Ding‘ wandelte. Als solches führte das Nest nicht einfach zu einer Naturalisierung, sondern bezeichnete einen familiären natural-sozialen Zwischenraum, der zunehmend zum Ziel weiblich konnotierter Sorge-Arbeit wurde.

## Abstract (en)

Today, many pregnancy guides mention a nesting instinct. According to this, pregnant women would be seized by an urge to create the right environment for their child, for example to buy baby equipment or clean the apartment. The concept of the nesting instinct forms a specific configuration of knowledge: While it is widespread in the popular field, it occupies a marginal position in the scientific field. In this paper, I will investigate the historical epistemology of this form of knowledge. In so doing, the following questions are addressed: How did the knowledge about a nesting instinct during pregnancy form? How was the nest as a specific natural-anthropogenic environment constructed? And to what extent do notions of gender and environment interact here? To answer these questions, the study takes the perspective of a history of knowledge in transit, in the longue durée from the nineteenth century to the present. The investigation reveals a gradual feminization of the concept of environment in the knowledge of the nesting instinct. Whereas in the nineteenth century it was often considered a male behavioral pattern and the nest was an analogy to the house, in the first decades of the twentieth century, the instinct transformed into a primarily female characteristic, with the nest representing the interior of the home. A decisive condition for this circulation of knowledge was that the nest became a ‘metaphorical thing’. As such, the nest did not simply lead to naturalization, but denoted a natural-social in-between space that increasingly became the goal of female care work.

## Das Wissen zum Nestbauinstinkt: Einleitende Bemerkungen

Spätestens kurz vor der Geburt soll es soweit sein – so die aktuelle Ratgeberliteratur: Die Schwangere spüre einen unwiderstehlichen Drang, ein Nest zu bauen, um damit die passende Umwelt für ihr Kind zu gestalten. Sie kaufe Möbel und Babykleidung, streiche Wände, dekoriere und putze. Zahlreiche Ratgeberbücher bezeichnen dieses Verhaltensmuster als „Nestbauinstinkt“ oder „Nestbautrieb“. So erfährt die Leserin eines Schwangerschaftsratgebers von den Autor_innen, die sich – in relativ typischer Kombination für das Genre – aus einer Mutter und einem Facharzt für Frauenheilkunde zusammensetzen, Folgendes: „Wenn Sie jetzt einen ungewöhnlichen Putzfimmel bekommen und alles schön sauber machen wollen, die Wohnung putzen, die Gardinen waschen wollen und das vorbereitete Kinderzimmer zum x‑ten Mal umräumen, dann könnte dies ein Hinweis auf den Nestbauinstinkt sein, der sich kurz vor der Geburt bei vielen Frauen einstellt“ (Grünebaum & Okko 2010: 279). Zurückgeführt wird dieses Phänomen meist auf Schwangerschaftshormone, wobei als zusätzlicher Beweis für seinen biologischen Ursprung gern auch auf ähnliche Prinzipien in der Tierwelt verwiesen wird. Beispielhaft erklärt ein Buch: „Gegen Ende der Schwangerschaft überflutet uns unser Körper nochmals mit aktivierenden Hormonen und wir sind dem Tierreich näher, als uns vielleicht bewusst ist, denn auch Tiere richten sich instinktiv ihr Nest oder ihren Bau her, um das Umfeld für die Ankunft des Nachwuchses sicher und komfortabel zu gestalten“ (Rainer-Trawöger 2017: ca. 276).

Das Konzept des Nestbauinstinkts kursiert heute vor allem in der zeitgenössischen Ratgeberliteratur, in der Expert_innen – deren Status über ihre Erfahrung, Profession oder wissenschaftlich-medizinische Kenntnis legitimiert wird – dieses Wissen an Laien weitergeben und auf diese Weise mit zu spezifischen Subjektformationen und Selbstverhältnissen beitragen (Maasen 2012). In geringeren Umfang finden sich Aussagen zum Nestbauinstinkt mitunter in Lehrbüchern für angehende Mediziner_innen (z. B. Buddeberg 2004: 143) und Hebammen (z. B. Mändle & Opitz-Kreuter 2007: 135). In den letzten Jahren wurde das Phänomen schließlich auch zum wissenschaftlichen Objekt, postulierte eine psychologische Studie (Anderson & Rutherford 2013) doch ein evolutionär bedingtes Nestbauverhalten bei Schwangeren, die ebenfalls auf Hormone und analoge Verhaltensweisen bei Tieren verwies. Bislang allerdings fand besagte Studie in der biomedizinischen und psychologischen Fachliteratur relativ wenig Beachtung: Sie wurde kaum zitiert.^1^ Allerdings wurde die Studie bald in Ratgeberliteratur aufgegriffen, um die Existenz eines Nestbauinstinkts nun zusätzlich über vermeintliche wissenschaftliche Evidenz zu belegen (z. B. Howland 2017: 250). Beim Nestbauinstinkt handelt es sich also um eine spezifische Wissensform, die aktuell im populären Bereich verbreitet ist und sich zwar durch das Rekurrieren auf den Bereich der Naturwissenschaft und Medizin legitimiert, dort jedoch derzeit keine nennenswerte Forschung zu dem Thema existiert. Dieser Beitrag setzt sich mit der Geschichte und den Möglichkeitsbedingungen dieses Wissens auseinander und ist dabei an drei Hauptfragen orientiert: Wie konstituierte sich das Wissen um einen Nestbautrieb in der Schwangerschaft in seiner spezifischen Form? Auf welche Weise wurde dabei das Nest als spezifische natural-anthropogene Umwelt produziert? Und inwiefern interagieren hier Vorstellungen von Geschlecht und Umwelt?

Mit seiner Fragestellung behandelt der Aufsatz ein Forschungsdesiderat zu diesem Thema. Nicht zuletzt auf Grund von Barbara Dudens (1987; 1993) einschlägigen historischen Untersuchungen existiert mittlerweile eine Vielzahl an kultur- und wissenschaftsgeschichtlichen Arbeiten zu Schwangerschaft und Geburt, die durch wichtige sozialwissenschaftliche und ethnologische Beiträge ergänzt werden.^2^ Trotz dieser Vielfalt an Literatur wurde das Wissen zum Nestbauinstinkt in der Schwangerschaft nicht behandelt oder allenfalls gestreift. Kürzlich hat sich allein Arianne Shahvisi (2020) mit dem Thema in einem Aufsatz aus Perspektive der Medizinethik und Geschlechterforschung auseinandergesetzt. Auch Shahvisi betont die Diskrepanz zwischen breit postuliertem Wissen zum Nestbautrieb einerseits und fehlenden wissenschaftlichen Forschungspraktiken andererseits. Eine historische Analyse des Nestbauinstinkts fehlt bislang noch.

Um einen Überblick über grundlegende historische Formationsprinzipien zu erarbeiten, soll der Nestbauinstinkt in der Schwangerschaft im vorliegenden Artikel aus einer Perspektive der *longue durée* (Grote 2015; Guggenheimer et al. 2013) betrachtet werden. Im Hintergrund dieser Geschichte stehen drei Thesen: Erstens soll gezeigt werden, dass das Wissen zum Nestbauinstinkt – wie auch Shahvisi argumentiert – vergeschlechtlicht war, die Art der Vergeschlechtlichung jedoch unterschiedlichen historischen Konjunkturen unterworfen war. Dabei erfolgte durchaus eine Essentialisierung von zeitgenössischen Geschlechternormen durch die Verwendung eines biologisch-determinierenden Erklärungsmodells. Solche Biologisierungen, die bei Geschlechtsvorstellungen oft auftreten, sind allerdings nicht unbedingt mit einer einfachen Naturalisierung gleichzusetzen. Zumindest im Falle des Nestbautriebes folgen sie komplexeren Mustern. Denn zweitens möchte ich das Konzept des ‚Nestes‘ mit der Denkfigur ‚Umwelt‘ in Bezug setzen. Zwar bezeichnet das Konzept des Nestes primär ein von der Kultur unbeeinflusstes, vor allem tierisch geschaffenes Objekt und scheint so zunächst zum Bereich der ‚Natur‘ zu gehören, auf den sich entsprechend Naturalisierungsprozesse beziehen. Es bildet jedoch keine vermeintlich unberührte und ‚wilde‘ Natur, sondern eine artifizielle, bereits ‚kultürliche‘ Natur, eine Umgebung, die sich ein Tier selbst geschaffen hat. Geht man von der Dichotomie von Individuum und Welt bzw. Innen und Außen aus, so nimmt das Nest eine Zwischenposition ein, es markiert ein ‚Drittes‘: Ähnlich wie beim Modell des Milieus (Canguilhem 1974), schafft sich ein Organismus hier seine eigene Umwelt, die eine Vermittlungsposition zur Außenwelt besitzt. Die Geschichte des Nestbautriebes spiegelt so teilweise auch die Geschichte von Umweltvorstellungen wider.

Und drittens soll meine Untersuchung zur historischen Epistemologie populären Wissens beitragen, indem ich die analytische Kategorie eines ‚metaphorischen Dinges‘ einführe. Dabei verwende ich den Begriff der historischen Epistemologie Hans-Jörg Rheinbergers Überblicksdarstellung (2017) folgend, im Sinne der ‚angelsächsischen Tradition‘ – also in einem erweiterten Sinne, der nicht nur nach der Geschichte der Wissenschaften fragt, sondern nach den historischen Möglichkeitsbedingungen von Erkenntnis und Wissen. Viele Abhandlungen zur historischen Epistemologie beschäftigen sich vor allem mit der wissenschaftlichen Sphäre (Feest & Sturm 2011). Dagegen ist für den Nestbauinstinkt eine Wissensgeschichte (Landwehr 2007; Speich Chassé & Gugerli 2012) angemessener. Schließlich liegt mit dem Nestbauinstinkt ein Wissenstypus vor, der zumindest heute vor allem in der populärwissenschaftlichen Sphäre zirkuliert und dessen Formation so von anderen Dynamiken und Bedingungen geprägt sein mag als primär wissenschaftliche Objekte. Ausgehend von der rezenten Quellenlage zu dem Phänomen soll hier – trotz der fundierten Kritik an der Trennung des ‚Populären‘ und des ‚Wissenschaftlichen‘ innerhalb der Wissenschaftsgeschichte (Secord 2004; Topham 2009) – aus heuristischen Gründen an dieser Unterscheidung festgehalten werden. Ihre Definition ist an Lesepublikum und Verbreitungsgrad orientiert. Während sich ‚populäres Wissen‘ in Texten findet, die oft eine hohe Auflage besitzen und sich an ein diverses, oft laienhaftes Publikum richten, adressiert ‚wissenschaftliches Wissen‘ stärker ein akademisches Fachpublikum und ist an spezifische Evidenzpraktiken gebunden. Zugleich ist die Unterscheidung zwischen den Wissenstypen nicht essentialistisch zu verstehen und erfolgt im Bewusstsein ihrer historischen Kontingenz. An der Geschichte des Nestbautriebes möchte ich zeigen, dass es sich dabei um kein isoliertes wissenschaftliches Konzept handelte, das sich in Alltagsdiskursen lediglich popularisierte, sondern dass sich dieses Wissen im Transit zwischen verschiedenen akademischen Disziplinen sowie zwischen wissenschaftlichen und populären Wissenskulturen formierte und modifizierte. Dabei waren nicht nur unterschiedliche Techniken und Praktiken wissenschaftlicher Forschung von Bedeutung – etwa Naturbeobachtungen oder Experimentalsysteme –, sondern ganz besonders veränderte soziokulturelle Bedingungen. Letztere waren für das Konzept in stärkerem Ausmaß prägend, weil das ‚Nest‘ nicht nur zum ‚epistemischen Ding‘ wurde, sondern zunehmend auch zu einem ‚metaphorischen Ding‘. Es transportierte so einen Überschuss an kulturellen und sozialen Bedeutungen mit, der den Austausch zwischen den Wissensfeldern erleichterte und eine breite Zirkulation möglich machte.

Der Zeitraum meiner historisch epistemologischen Analyse reicht von den frühen 2010er Jahren bis zur Formation neuzeitlichen Wissens über Schwangerschaft zurück, also bis zum ausgehenden 18. Jahrhundert, wobei ein Schwerpunkt auf das 20. Jahrhundert gelegt wird. Die Untersuchung des Wissens zum Nestbautrieb stützt sich auf heterogenes Quellenmaterial in deutscher und englischer Sprache. Das methodische Vorgehen bei der Auswahl des Materials erfolgte auf heterogene Weise: durch die Recherche in geschichts- und kulturwissenschaftlicher Sekundärliteratur zur Geschichte der Schwangerschaft und Kategorisierungen des Psychischen; durch die Lektüre von einflussreichen geburtshilflichen Lehrbüchern und Schwangerschaftsratgebern; und durch eine Stichwortsuche nach englisch- und deutschsprachigen Begrifflichkeiten um den Nestbau in digitalen Portalen.^3^ In Bezug auf ihre inhaltliche Ausrichtung lassen sich die so gesammelten Quellen in zwei Untergruppen unterteilen: Zum einen gehören dazu Texte, die sich vorwiegend mit menschlichem Verhalten und Erleben in Bezug auf Schwangerschaft beschäftigen. Hierzu zählen geburtshilfliche und gynäkologische Lehrbücher sowie populäre Ratgeber, die sich über schwangere Gefühle und psychische Zustände äußern, Studien der Psychiatrie und Psychologie, Zeitschriftenartikel und mediale Darstellungen. Zum anderen wird Wissen zum Nestbautrieb herangezogen, das sich schwerpunktmäßig mit tierischem Verhalten beschäftigt. Dabei handelt es sich um Studien aus der Tierpsychologie, den Verhaltenswissenschaften und der Ethologie ebenso wie um einige populäre Darstellungen der Natur und Tierwelt. Für den untersuchten Zeitraum wurden zwei Hautphasen der Vergeschlechtlichung identifiziert – zunächst eine Formation des Nestbauinstinktes als primär männliches Attribut im 19. Jahrhundert und anschließend eine ‚Verweiblichung‘ des Konzepts im 20. Jahrhundert, das schließlich mit einer Engführung auf menschliche Schwangerschaft in der zweiten Jahrhunderthälfte einherging.

## Das männliche Nest – Zum Nestbauinstinkt im 19. Jahrhundert

Im 18. und 19. Jahrhundert variiert das Wissen zu Nestbautrieb in den zwei unterschiedlichen Quellengruppen stark. In der Literatur des 18. und 19. Jahrhundert zu Schwangerschaft und Geburt findet sich die Vorstellung kaum. Vereinzelt ist hier aber vom Begriff des Nests zu lesen, der primär als Metapher für das Geburtslager verwendet wurde. So empfahl etwa der Leipziger Mediziner Carl Hennig (1873) in seinem Frauenratgeber, die Schwangere solle sich ein gut stehendes Bett zur Niederkunft herrichten, ähnlich wie etwa auch Rehe zum Gebären das Unterholz aufsuchten oder Vögel ein Nest bauten. Ein ähnliches Verständnis vom Nest als Bettstelle oder Lager ist teilweise auch in den von Kira Jürjens im gleichen Heft analysierten Einrichtungsratgeber des ausgehenden 19. Jahrhundert zu finden. Dagegen waren heutige Vorstellungen eines Nestbautriebes, wie er in den eingangs zitierten Beschreibungen des vorliegenden Artikels vorkommt, nicht zu finden. Und auch das breitere Spektrum an mütterlicher Emotionalität, das heute für die Schwangerschaft üblich ist, fehlte bei der Beschreibung aus dieser Zeitperiode (Malich 2017). Die blieb noch in den einschlägigen Quellen der ersten Hälfte des 20. Jahrhunderts unverändert. Zwar hatten sich spätestens um 1900 Modelle des Pränatalen und einer komplexen maternal-fötalen Beziehung formiert (Arni 2012) und in Diskursen tauchte nun mitunter bereits ein „Mutterschaftsgefühl“ (Siegel 1919: 203) bei der Schwangeren auf (Malich 2016), Vorstellungen eines Nestbautriebs bildeten in den ersten Jahrzehnten des 20. Jahrhunderts aber weiterhin eine Leerstelle. Für die menschliche Schwangerschaft schien dieses Phänomen also lange Zeit noch keineswegs zum festen Repertoire der psychischen Erscheinungen zu gehören.

Dagegen kommt der Begriff des Nestbautriebs in der zweiten Quellengruppe vor, nämlich bei Beschreibungen der Tierwelt. Im 19. und frühen 20. Jahrhundert formierten und verbreiteten sich die Felder der vergleichenden Verhaltensforschung, der Tierpsychologie, der Zoologie und der Verhaltensbiologie. Zu ihren bevorzugten Praktiken der Wissensgenerierung gehörte die Beobachtung von Tieren, die Bestandteil akademisch-zoologischer Forschung war und ebenso eine beliebte Beschäftigung des gebildeten Bürgertums darstellte (Schmoll 2004). Allgemein erfolgte in vielen westlichen Ländern die Grenzziehung zwischen ‚Populärwissenschaft‘ und ‚Wissenschaft‘ im Laufe des 19. Jahrhunderts und variierte in ihrer Form (Bensaude-Vincent 2009). Gerade für die oben genannten disziplinären Felder im Umkreis der Zoologie war der fließende Übergang zwischen sogenanntem Amateur- und Expertentum charakteristisch (Taschwer 2002). Teilweise erfolgten die Tierbeobachtungen dabei im Rahmen einer breiteren Heimatkunde, die dazu beitrug, regionale bzw. nationale Identität auszubilden und die gegen Ende des 19. Jahrhunderts teilweise in frühe Naturschutzbewegungen abzweigte. Hierbei formten sich Vorstellungen einer den Menschen umgebenden natürlichen ‚Umwelt‘, wenngleich diese zunächst jedoch oft eher aus einer romantisierten und deutsch-nationalisierten „Museumsnatur“ bestand (Schmoll 2004: 232).

Besonders gut boten sich Vögel zu tierischen Verhaltensstudien an, da sie sich relativ einfach beobachten ließen. Auf diesem Wege unternahmen die Vögel im Laufe des 19. Jahrhunderts eine „Wanderung aus dem Kochtopf mitten in die Herzen“, was schließlich in der Etablierung einer Vogelschutzbewegung als Teil des Naturschutzes kulminierte (Schmoll 2004: 294). In seinem populären Buch *Das Leben der Vögel *rühmte etwa der Zoologe und Naturschriftsteller Alfred Brehm: „Ich zähle die Beobachtung der Vögel beim Nestbau und Brutgeschäft zu den schönsten Freuden des Forschers und Naturfreundes“ (1861: 242). Einer jener frühen Vogelbeobachter war Constantin Wilhelm Lambert Gloger, der zeittypisch zugleich als Forscher, Naturfreund sowie Herausgeber ornithologischer Fachpublikationen und ‚gemeinnütziger‘ Schriften mit breiterem Publikum auftrat. Schon zuvor war der Nestbautrieb von einigen Gelehrten wie dem dänischen Zoologen Friedrich Faber eher en passant dokumentiert worden und als „Trieb“, der vor allem „bey den Vögeln“ aber „nur sporadisch bey den Säugethieren“ gefunden werde definiert worden (Faber 1826). Für Gloger stellte nun aber gerade der Nestbautrieb von Vögeln einen relevanten und eigenständigen epistemischen Gegenstand dar. Von ihm stammt einer der ersten wissenschaftlichen Aufsätze, die sich schwerpunktmäßig mit diesem Phänomen beschäftigen (Gloger 1854). Unter dem Titel „Der Nestbau-Trieb mancher unbeweibten Vogel-Männchen“ gibt er einen Überblick aus unterschiedlichen Beobachtungsstudien zu diesem „Instincte“, welcher unter anderem bei den Webervögeln, der Beutelmeise oder dem europäischen Zaunschlüpfer dokumentiert worden sei (Gloger 1854: 375). Neben Vögeln gerieten auch andere Tierarten ins Visier der Verhaltensforschung und Zoologie. Hier beschrieben etwas später einige andere Autoren den Nestbauinstinkt, insbesondere von Insekten und Fischen, etwa Ameisen (Möller 1898) oder Stichlingen (Matthes 1861). Auf diese Weise stieg in den Beobachtungswissenschaften des 19. Jahrhunderts der Nestbauinstinkt als Forschungsobjekt auf.

Schon bald war der Nestbautrieb nicht mehr nur ein sich selbst genügender epistemischer Gegenstand, den es galt, möglichst akkurat zu erfassen. Sondern er diente als Mittel zum Zweck, um ein übergeordnetes epistemisches Ding – das Wesen des ‚Instinkts‘ an sich – zu verstehen. Dazu trug der berühmte Privatgelehrte Charles Darwin in seinem 1859 erstveröffentlichten *On the Origin of Species *bei: In dem Kapitel zu ‚Instinct‘ unterschied er diesen von der ‚Gewohnheit‘ (Darwin 1860: 217–253). Anders als diese, folge der Instinkt dem Prinzip der Erblichkeit. Als Beispiele für erbliche Instinkte führte Darwin das identische Nestbauverhalten der Vogelmännchen sowohl des amerikanischen als auch des europäischen Zaunkönigs an, welches er auf einen erblichen Instinkt zurückführte.

In der Nachfolge von Darwins Werk bemühte sich ein Teil des Forschungsfeldes um eine genauere Definition des Instinkts, wobei der Nestbautrieb als beliebtes Erkenntnisinstrument diente. Dazu gehörten etwa der britische Naturforscher Alfred Russel Wallace und, einige Jahrzehnte später, der britische Tierpsychologe Conwy Lloyd Morgan (Boakes 1984). Beide postulierten, dass das Nestbauverhalten stärker als von Darwin behauptet von Gewohnheit bzw. Lernen geprägt sei. Gerade gegen Ende des 19. Jahrhunderts ist dabei eine Abkehr von der reinen Tierbeobachtung zu verzeichnen. Vielmehr wurden dabei nun auch Experimentalsysteme mit Vögeln als Versuchstieren eingesetzt, in denen versucht wurde, deren Nestbauverhalten zu manipulieren, etwa indem sie als Jungvögel von ihren Eltern isoliert wurden, sodass sie das Nestbauen nicht lernen konnten. Das Nestbauen wurde hier so zu einem Schlüsselmoment zeitgenössischer Debatten darüber, ob und inwieweit Instinkte erlernt werden könnten und was ein ‚echter‘ Instinkt sei. Zugleich nahm das Nest hier eine Zwischenposition zwischen natürlicher Anlage und kultürlichem Lernprozess ein.

Zeitgenössische Vorstellungen des Nestes waren auch von Geschlechternormen geprägt. Zwar gingen weder Faber noch Brehm in ihren Büchern zum Vogelleben nennenswert auf Geschlecht ein, wenn sie über das Nestbauen schrieben. Stattdessen widmen sie viele Seiten einer akribischen Beschreibung und Bewertung der Nester unterschiedlicher Vogelarten – ihrer Form und Beschaffenheit, vor allem aber ihrer Schönheit, Sorgfalt und Kunstfertigkeit. Wird aber in dieser Periode ein Geschlecht der bauenden Tiere hervorgehoben, so ist es das männliche. Darwin beschreibt Zaunkönigmännchen. Die von Matthes erwähnten nestbauenden Stichlinge sind männlich. Und Gloger beschäftigt sich in seinem Themenaufsatz sogar schwerpunktmäßig mit dem Nestbautrieb von Vogelmännchen – wohl auch, weil der den von den Männchen gebauten Nestern die höchste Qualität bescheinigte. Denn ihm gemäß handle es sich hierbei ausschließlich um solche „Gattungen, in deren Instincte die Neigung und Fähigkeit liegt […] ganz besonders künstliche Nester zu bauen“ (1854: 375). Hierbei ist der Begriff der ‚Künstlichkeit‘ eher im Sinne der ‚Kunstfertigkeit‘ zu verstehen, sodass sich schon in dieser Beschreibung zweierlei Vorstellungen finden lassen: Einerseits illustriert sie erneut die ambivalente Konstruktion des Nestes als eine Art ‚künstlich‘ oder ‚künstlerisch‘ geschaffene und zugleich natürliche Umwelt. Andererseits lenkt der Begriff der Künstlichkeit den Fokus auf die Bautätigkeit und ihr Produkt. Darin klingen Ideen von Schaffenskraft an, die in der Geschlechterkultur des 18. und 19. Jahrhunderts maskulin kodiert waren. Auch in anderen Formulierungen Glogers scheinen Attribute durch, die dem zeitgenössischen männlichen ‚Geschlechtscharakter‘ (Hausen 1976) des Bürgertums entsprachen und die Aktivität, Stärke, Beharrlichkeit und Rationalität beinhalten. So schenkte Gloger den tierischen „Baukünstlern“ viel Bewunderung, indem er beschrieb, dass etwa die männlichen Beutelmeisen durch „schwierige Arbeit […] wahrhaft künstlerische Bauten“ errichteten (1854: 376–378). Andere Arten würdigte er dafür, wie sie „mit unermüdlichem Fleisse“ und in „schlauer Weise“ ihre Nester bauten (ebd.). Beim nordamerikanischen Zaunkönig macht der Autor seinen Bezugsrahmen der bürgerlichen Menschenwelt explizit. Dieses Vogelmännchen zeige zwar eigentlich viel „Theilnahme für seine Gattin“, für deren künftige Zufriedenheit es seine „Wohnung ohne Beihilfe“ baue (Gloger 1854: 377). Allerdings komme es bei dieser Vogelart erst nach dem Nestbau zur Paarbildung. Deshalb sei der Erfolg des Männchens unsicher, da die Wohnung „seiner Hausfrau […] nicht immer ausreichend zusagt“ (ebd.). Diese Metaphern aus der Menschenwelt – die Begriffe der Wohnung, der Gattin und der Hausfrau – sind keineswegs zufällig, haben doch historisch-epistemologische Fallstudien gezeigt, dass Metaphern wissenschaftliche Forschungsprogramme prägen und so einen geradezu ontologischen Status erreichen können (Brandt 2004). Die metaphorische Sprache macht deutlich, dass die Augen, die mit ihrer Beobachtung der Vogelwelt Wissen generierten, von bürgerlichen Werten und der zeitgenössischen Geschlechterordnung der Menschenwelt geprägt waren. Hier war es der Mann, der die ‚Wohnung‘ baute und besaß – sodass an dieser Stelle der Begriff des ‚Nestes‘ im Sinne des gesamten Hauses (statt als Synonym für Inneneinrichtung) zu verstehen ist. Die künftige ‚Hausfrau‘ und ‚Gattin‘ war diejenige, die dort einzog (oder nicht). Darin spiegeln sich vergeschlechtliche Vorstellungen von Besitzverhältnissen, die unter anderem im Familienrecht des Bürgerlichen Gesetzbuchs von 1896 enthalten waren, in dem der Ehemann die Entscheidungsgewalt über Wohnort hatte (Meder et al. 2010). Das Nestbauen war im 19. Jahrhundert also nicht nur zum ‚epistemischen Ding‘ der Beobachtungswissenschaften geworden – er trat zugleich auch als ‚metaphorisches Ding‘ in Erscheinung, in dem sich Werte und Normen der bürgerlichen Gesellschaft widerfanden.

Insgesamt war der Nestbautrieb also ein wiederkehrendes Motiv in Ornithologie, Tierpsychologie und Evolutionstheorie des 19. und frühen 20. Jahrhunderts, wo er oft im Grenzbereich akademischer und populärer Diskurse auftauchte. Für dieses frühe Wissen zum Nestbautrieb sind drei Punkte von Bedeutung: Erstens handelte es sich bei den beobachteten und untersuchten Tieren primär um Vögel, teilweise auch um Insekten und Fische – dagegen nicht, oder noch kaum, um Säugetiere. Zweitens erfolgte bereits eine teilweise Essentialisierung des Nestbautriebes als ‚Trieb‘ oder ‚Instinkt‘, der speziesübergreifend wirksam war und körperliche Ursachen hatte. Hinsichtlich des körperlichen Erklärungsmodells standen generell Debatten um Erblichkeit im Vordergrund. Wenn es um spezifisch körperliche Auslöser des Triebes ging, so wurden außerdem mitunter nervöse Mechanismen erwähnt. Und drittens wurde das Nestbauverhalten beiden Geschlechtern zugesprochen, wobei häufig der Nestbau durch Männchen im Vordergrund stand.

## Das Nest des Tierweibchens – 1930er bis 1960er Jahre

Zu Beginn des 20. Jahrhunderts wanderten Vorstellungen des Nestbautriebes in andere, sich damals formierende Wissensfelder. Zwar blieb der Nestbauinstinkt ein zentrales Forschungsobjekt der Ornithologie. Das Wissen wurde nun aber auch Bestandteil der aufstrebenden Ethologie – also der tierischen, artspezifischen Verhaltensforschung – ebenso wie der entstehenden Endokrinologie – der Lehre der endokrinen Drüsen bzw. der Hormone. Hierbei blieben insbesondere in der Ethologie Praktiken aus den Beobachtungswissenschaften zentral, wohingegen die Endokrinologie stärker von Experimentalsystemen geprägt war. In den neuen Feldern bezog sich der Begriff weiterhin auf tierisches Verhalten. Doch die drei oben genannten Aspekte zum Wissen des Nestbautriebes veränderten sich in der Zeitperiode von den 1930er bis in die 1960er Jahre.

Zunächst zur Ethologie, zu deren zentralen Figuren Konrad Lorenz gehört (Burkhardt 2005). Die Instinkttheorie hatte nach dem Ersten Weltkrieg zwischenzeitlich ihre akademische Reputation verloren, weil einige führenden Psychologen sie als zu verallgemeinerndes Konstrukt zurückgewiesen hatten (Griffiths 2008). Durch Lorenz erlebte die Instinktlehre ein Revival, als er sie Mitte der 1930er Jahre in seinem ‚psychohydraulischen Instinktmodell‘ reformulierte. Für das Modell diente ihm das Nestbauverhalten von verschiedenen Vögeln als wichtiges Anschauungsmaterial (z. B. Lorenz 1937). Im hydraulischen Modell war der Instinkt als eine Art inneres Reservoir zu denken. Das Reservoir steigt durch nervöse und hormonelle Mechanismen langsam an und kann durch einen externen Reiz ausgelöst werden, wenn dieser eine gewisse Schwelle überschreitet. Je voller das Reservoir, desto niedriger die Reizschwelle, bis hin zu ‚Leerlaufhandlungen‘ – also etwa Nestbaubewegungen ohne vorhandenen Nistplatz. Lorenz rekurrierte in diesem Zusammenhang meist auf Vögel allgemein und betonte weder unterschiedliche Arten noch geschlechtsspezifische Merkmale.

Eine spezifische Vogelart bekam durch Lorenz jedoch ganz besondere Aufmerksamkeit, ja, wurde geradezu eine öffentliche Berühmtheit: die Graugans, die Lorenz als zentrales Studientier diente. In Bezug auf den Nestbau fiel hierbei die Wahl genau auf ein Tier, bei dem eine strikte Geschlechtertrennung beobachtbar ist: In dieser Gattung baut das Weibchen das Nest, das Männchen dagegen verteidigt es gegen Angreifer. Nur das Graugansweibchen brütet die Eier aus – und rollt diese mit spezifischen Eirollbewegungen zurück ins Nest, wie Lorenz und sein Kollege Nikolaas Tinbergen in einer einflussreichen Studie dokumentierten (Lorenz & Tinbergen 1938). Und auch einige andere Eigenschaften der Graugans entsprechen wohl der idealtypischen Geschlechterordnung aus den 1930er Jahren. So rühmte schon Lorenz’ Lehrer, der Zoologe Oskar Heinroth, die lebenslange Treue eines Grauganspaars. Lorenz konservative gesellschaftspolitische Vorstellungen und seine Nähe zu nationalsozialistischen Positionen sind mittlerweile bekannt (Föger & Taschwer 2001). In seiner Wahl des Studientieres Graugans spiegelten sich wohl traditionelle Ehe- und Geschlechternormen – zugleich verfestigte das so hervorgebrachte Wissen diese Normen.

Lorenz hatte nicht nur Einfluss auf wissenschaftliche Dynamiken, sondern auch auf eine breitere Öffentlichkeit. Denn er suchte früh die Nähe zur populären Sphäre (Taschwer 2002). Schon Mitte der 1930er Jahre hielt Lorenz Vorträge in den volkstümlichen Hochschulkursen der Universität zu Wien, wo er seinem Publikum bereits Tier-Mensch-Homologien präsentierte, die in seinen primär wissenschaftlichen Publikationen der Zeit noch fehlten. Vor diesem Hintergrund wurden seine Verhaltensbeschreibungen der Graugans nicht nur innerhalb der Ethologie diskutiert, sondern parallel auch früh breiter bekannt. Im Film zur Ethologie der Graugans, den er von 1935 bis 1937 drehte und der 1950 offiziell erschien, waren Aufnahmen nestbauender und brütender Graugansweibchen zu sehen.

Für die Geschichte des Nestbautriebs haben Lorenz Arbeiten zweierlei Bedeutung. Erstens führte die frühe Popularität ethologischer Werke zu einer Verbreitung des Wissens zum Nestbautrieb. Zweitens lässt sich hier durch die Wahl des Studientiers und damit der forschungstechnischen Infrastruktur eine erste ‚Verweiblichung‘ dieses Instinkts feststellen. Zwar gab es in dieser Zeit durchaus weiter ornithologische oder zoologische Forschung zu einem neutral oder männlich verstandenen Nestbauinstinkt. In Lorenz’ Arbeiten zur Graugans wurde der Instinkt aber dem Vogelweibchen zugeordnet und an ‚mütterliche‘, hütende und pflegende Tätigkeiten gekoppelt.

Die Verweiblichung des Triebes – und, noch spezifischer, die Verknüpfung mit Schwangerschaft – intensivierten endokrinologische Forschungssettings. Schon Lorenz (z. B. 1937) nannte Hormone als somatische Auslöser für die Instinktmotivation. Die Verknüpfung des Nestbauinstinkts mit Schwangerschaftshormonen erfolgte aber in einem anderen Feld, nämlich in der medizinischen Forschung zum frühen Schwangerschaftstest, wobei zwei englischsprachige Publikationen aus Großbritannien und den USA zentral sind. 1933 erschienen zwei experimentelle Studien, eine des Physiologen Bertold Wiesner mit seiner Kollegin Norah Sheard (1933) und eine der Medizinerin Esther Bogen Tietz (1933). Interessanterweise entstanden beide Studien als eine Art ‚Nebenprodukt‘ medizinischer Technologien. Die Autor_innen beider Studien sollten sich in ihrer Forschung nämlich in erster Linie mit der Weiterentwicklung des frühen Schwangerschaftstests beschäftigten. Dabei handelte es sich um einen endokrinologischen Test, der 1927 in Berlin von den beiden Medizinern Selmar Aschheim und Bernhard Zondek konzipiert worden war.^999^ Bei dem Test handelte es sich damals noch um ein Bioassay, also eine Untersuchung an lebendem Gewebe, wobei der Urin von Frauen in Versuchstiere gespritzt wurde (Olszynko-Gryn 2014). Während in der ursprünglichen Testversion Mäuse zum Einsatz kamen, arbeite Tietz an der Entwicklung eines Schwangerschaftstests mit Hasen, Wiesner und Sheard mit Ratten. In diesen drei genannten Varianten des Tests wurden ausschließlich Weibchen verwendet. Wenn eine Schwangerschaft vorlag, sollten sich die tierischen Geschlechtsorgane nach bestimmten Kriterien verändern. Dazu wurden die Tiere getötet und seziert. Davor benutzten Tietz, Wiesner und Sheard ihre noch lebenden Versuchstiere auch für wissenschaftliche Zusatzstudien zu anderen Themen – sie verwandelten also die diagnostischen Systeme temporär zurück, in Experimentalsysteme, so dass sich einige der ‚technischen Dinge‘ zurück in ‚epistemische Dinge‘ wandelten (Rheinberger 1992). Hierbei gaben die verwendeten Materialien – ausschließlich weibliche Versuchstiere und Urin von Frauen – jedoch bereits viele Parameter für die Wahl geeigneter epistemischer Objekte vor.

Beide Gruppen entschieden sich für damals verbreitete Themen der Instinkte und endokrinologischen Geschlechterpsychologie. Letztere war spätestens durch die spektakulären Tierexperimente des Physiologen Eugen Steinach bekannt, die dieser ab 1912 in Wien durchführte. In diesen nahm er vermeintliche ‚Geschlechtsumwandlungen‘ durch Verpflanzungen endokriner Drüsen vor, durch die sich auch das Verhalten den gängigen Geschlechtsrollen gemäß verändern sollte (Stoff 2008). Für ihre Studien zu Instinkten nutzten Tietz, Wiesner und Sheard schlicht das bestehende Material und orientierten sich an den damals gängigen Praktiken der Schwangerschaftsdiagnostik: Sie spritzten den Tieren, wie gewöhnlich, zunächst den Urin schwangerer Frauen. Danach suchten sie nach Verhaltensmodifikationen, zu denen sie auch das Nestbauverhalten zählten. Beide postulierten unabhängig voneinander, dass ein Nestbauinstinkt vorlag und dass Geschlechtshormone – nicht, wie zuvor angenommen, neuronale Mechanismen – als Auslöser zu betrachten seien. Innerhalb dieses Kontextes erschien der Nestbauinstinkt (mit durch das begrenzte Forschungssetting vorgegeben) als weiblich und war zudem mit der Schwangerschaft verknüpft. In ihrem Buch *Maternal Behavior in the Rat* (1933) subsumierten Wiesner und Sheard den Trieb unter mütterlichem Verhalten. Hier erschien die Mutter somit als Verantwortliche für das Schaffen der geeigneten Umwelt ihres Kindes. Die Studien der 1930er Jahre beeinflussten in den folgenden Jahrzehnten psycho-endokrinologische Forschung zu mütterlichen Verhaltensmustern bei Säugetieren, zu denen nun auch der Nestbau gezählt wird (z. B. Rosenblatt 1990).

Das Wissen zum Nestbauinstinkt war also einerseits von den eben beschriebenen Kontingenzen der Testsysteme und der Forschungspraktiken geprägt und andererseits war es von populären Geschlechtsvorstellungen und gesellschaftlichen Normen beeinflusst. Insgesamt transformierten sich in den Feldern von Endokrinologie und Ethologie die drei Aspekte des Nestbauinstinkts in dieser Periode folgendermaßen: Erstens verengte bzw. verschob sich die Vielfalt der untersuchten Tiere. Weiter stand primär der Nestbauinstinkt von Vögeln, insbesondere von Gänsen, im Vordergrund. Daneben rückten nun jedoch immer mehr Säugetiere (wie Mäuse, Ratten und Hasen) ins Blickfeld. Zweitens veränderte sich das körperliche Erklärungsmodell des Instinkts: Anstatt nervöser Mechanismen schienen nun Hormone als Auslöser, und zwar oft spezifisch ‚weibliche Schwangerschaftshormone‘. Und drittens veränderte sich, mit den ersten beiden Punkten verbunden, die geschlechtliche Codierung des Instinkts, er feminisierte sich und wurde eine Eigenschaft weiblicher Tiere. Insbesondere wurde er nun dem mütterlichen Verhaltensmuster zugeordnet. In dieser spezifischen Form erfolgte die nächste Station der Zirkulation des Nestbauinstinkts.

## Der Nestbau der menschlichen Schwangerschaft

Ab den 1940er und 1950er Jahren wanderte das Wissen über den Nestbauinstinkt von Tier zum Mensch. Denn es fand Eingang in einen weiteren diskursiven Bereich, nämlich den der menschlichen Schwangerschaft. Nun wurde der Begriff des Nestbautriebes in schwangerschaftsbezogener Fachliteratur aus Psychologie, Psychoanalyse und Gynäkologie erwähnt. Hier bildeten die Referenzen auf Tiere, die Attribuierung als weiblich und das hormonelle Wirkmodell Kontinuitäten. Zugleich tauchte der Begriff jetzt in diesbezüglichen Diskursen oft eher nebenbei auf, um Verhaltensmuster von schwangeren Frauen zu charakterisieren, insbesondere in Bezug auf Hausarbeit. Als eine der ersten – möglicherweise sogar als Erste – behandelte die Psychoanalytikerin und Medizinerin Helene Deutsch den Trieb in ihrem Werk *Psychologie der Frau *(1954; bereits 1944/45 als *The Psychology of Women* publiziert). Hier beschrieb Deutsch die „‚nestbildende‘ Aktivität“ in der Schwangerschaft, die sie als „Ausdruck des Instinktes“ begriff und mit dem typischen Verhalten des „Tierweibchens“ verglich (1954: 125, 140). Für Deutsch bestand dieser Instinkt im „Bedürfnis, im grossen oder kleinen für das Erwartete zu bauen“, das verschiedene Formen annehmen könne: „Ein neues Haus, ein in Behaglichkeit und Schönheit perfektes Kinderzimmer, eine Babyausstattung oder ein kleines, selbstgestricktes Jäckchen“ (Deutsch 1954: 125).

Schon früh war auch vom Putzen in der Schwangerschaft die Rede. Der Gynäkologe Gerd Döring erklärte etwa auf einem Symposium der Deutschen Gesellschaft für Endokrinologie, er habe in einer Studie festgestellt, dass ein Drittel der Frauen kurz vor Menstruationsbeginn eine erhöhte „Antriebshaftigkeit“ zeigten, was er auf Veränderungen der Sexualhormone zurückführte (Döring 1958a: 44). Dies, so meinte er weiter, habe er auch bei einigen Schwangeren kurz vor Geburtsbeginn beobachtet, „so daß man von einer Art ‚verkümmerten Nestbautriebes‘ sprechen könnte“ (Döring 1958a). In einem im gleichen Jahr erschienenen Aufsatz spezifizierte Döring diese Antriebshaftigkeit: Er halte „größere außerplanmäßige Putzaktionen, große Wäsche und dergleichen“ von Schwangeren für eine solche „Art verkümmerten Nestbautriebes“ (Döring 1958b: 150). Wenngleich er nicht ausführte, warum der Trieb ‚verkümmert‘ sei, ist anzunehmen, dass die Charakterisierung implizit an Degenerationstheorien anschloss.

Was führte dazu, dass das Wissen zum Nestbau nun auf die menschliche Schwangerschaft ‚übersprang‘? Das eine, diskursverändernde Werk war es wohl nicht. Die Analyse der Quellen hat bislang keinen ‚Urtext‘ ergeben, durch dessen Rezeption der Begriff des Nestbautriebes in die Menschenwelt Eingang gefunden hätte. Vielmehr erfolgte der Rekurs auf das Nestbauen in den Quellen lediglich als Feststellung einer beobachteten Tatsache; weder Deutsch noch Döring nennen Referenzliteratur oder geben andere Belege an. Da beide aus der Medizin kamen, liegt nahe, dass sie zeitgenössische endokrinologische Modelle kannten. Auch ist zumindest im Fall von Helene Deutsch davon auszugehen, dass sie mit ethologischen Ideen vertraut war, lebte sie doch bis in die 1930er Jahre wie Lorenz in Wien und bewegte sich wie er in den dortigen akademischen Zirkeln (Stephan 1997). Das Fehlen von entsprechenden Zitationen in den Primärquellen macht es für die wissensgeschichtliche Analyse zwar schwerer, die genaue genealogische Herausbildung des menschlichen Nestbautriebs zu rekonstruieren. Zugleich legt es aber nahe, dass es sich hierbei um die Aufnahme fachfremden oder populären Wissens handelte, das für die Autor_innen als so selbstverständlich erschien, dass es keiner ausführlicheren Referenz bedurfte. Die Möglichkeitsbedingungen für die beginnende Integration des Nestbautriebes in die menschliche Schwangerschaft sind somit im kulturellen Kontext der zeitgenössischen Geschlechterordnung zu suchen, die von veränderten Weiblichkeitsnormen geprägt war. Diese Transformationen bündeln sich im parallelen Aufstieg einer Metapher: der des Nests.

Ab der zweiten Hälfte des 19. Jahrhunderts verbreitete sich der Begriff des Nests als Analogie für zwei verschiedene Formen der Umwelt, nämlich zum Heim der menschlichen Familie und zum weiblichen Körper. Erstens wurde das ‚Nest‘ in der zeitgenössischen Populärkultur zur dominanten Umschreibung für den Raum der Familie und des Privaten – Kinder sollten nun in einem gemütlichen ‚Nest‘ aufwachsen. Beispielsweise tauchte um 1880 in englischsprachiger Literatur der Begriff des „empty nest“ auf für Eltern, deren Kinder groß geworden waren (Schmidt 2020: 24). Schließlich wurde das Nest vor allem in den ersten Jahrzehnten des 20. Jahrhunderts zu einer zentralen Metapher. Wie im Beitrag von Jürjens beschrieben wird, war schon in Einrichtungsratgebern für die bürgerliche Hausfrau der Kaiserzeit vom Nest zu lesen, das die Frau herzurichten habe, worunter teilweise das Bettenlager, teilweise aber bereits das gesamte Interieur der Wohnung verstanden wurde. Anders als in den früheren Diskursen zum Nestbau der Vögel, in denen das Nest für die gesamte Wohnung bzw. das Haus stand, ging es hier eher um Bereiche des Innenraums. Das Nest fungierte als Art der ‚inneren Umwelt‘ für Ehepaar und Familienangehörige, die selbst gestaltet war und die den Übergang zur ‚äußeren Umwelt‘ vermittelte. In Zusammenhang mit der Metapher des Nestes als hygienisch gepflegter und ästhetisch gestalteter Innenraum entwickelte sich eine emotional gefärbte Verwendung des Begriffs, die sich nah an Konzepten der ‚Atmosphäre‘ aus Käthe von Boses Aufsatz bewegt. So mahnte etwa der Journalist Karl Stugau die „Hausfrau“ in einem Zeitungsartikel, auf „den Flaum im Nest“ zu achten, der nicht entstehe, wenn sie nur hart arbeite und sich pflichttreu zeige, sondern erst, wenn sie sich auch wie eine „liebevolle Gattin“ und „zärtliche Mutter“ verhalte, in deren „Haus […] allen Deinen Familien-Angehörigen wohl ist“ (1875: 1). Hier steht der Nestbau als Metapher für die Erzeugung von Wohlgefühl und Zärtlichkeit, also weiblich konnotierter Gefühlsarbeit.

Im frühen 20. Jahrhundert verbreitete sich die kombinierte Bedeutung des Nests im Sinne eines liebevollen und dekorativ gestalteten Heims für Ehemann und Kinder endgültig. Berühmt sind beispielsweise die seit 1913 publizierten *Nesthäkchen*-Kinderbücher von Else Ury, in denen das Nest für die bürgerliche Familienidylle steht. Die Bedeutung vom Nest als Familienheim war die Voraussetzung der Vorstellung eines weiblichen Nestbautriebes. Zugleich führte die semantische Ausweitung des Begriffs (als dekoriertes, sauberes Interieur und als Geborgenheit gebendes Familienheim) dazu, dass das ‚Nest‘ in dieser Periode zu dem wurde, was Hans Blumenberg (1997: 9) als „absolute Metapher“ bezeichnet: eine Metapher, die nicht mehr nur dem Vergleich oder der Ausschmückung dient, sondern die sich verselbstständigt hat und so die Wissensgenerierung zu der von ihr bezeichneten Idee (in diesem Fall: der familiären Umwelt) mit beeinflusst. Da sich in absolutes Metaphern auch „Leitvorstellungen für die Orientierung und das Verhalten in der Welt kristallieren“ (Brandt 2004: 31) bildete das Nest ein Einfallstor für zeitgenössische kulturelle Normen und Geschlechterrollen in wissenschaftliche Konzepte.

Zweitens bekam der Begriff des ‚Nests‘ genau in dieser Zeit einen Ort im Körperinneren der Frau und zwar zunächst durch die Terminologie innermedizinischer Diskurse. Denn hier setzte sich in den ersten Jahrzehnten des 20. Jahrhunderts die Bezeichnungen der ‚Nidation‘ (Einnistung) als Teil der Embryonalentwicklung durch, womit der Uterus zum Nest wurde. Bereits im Verlauf des 18. und 19. Jahrhunderts hatte sich der medizinische Blick auf Reproduktionsvorgänge und den schwangeren Körper intensiviert. So entstand in verschiedenen embryologischen und geburtsmedizinischen Wissensfeldern ein Entwicklungsmodell fötalen Wachstums (Hopwood 2000; Wülfingen et al. 2015). Schließlich wurde die Schwangerschaft in diesem Zeitraum ein medizinisches Modell dafür, „was die Umwelt vermag“, indem die Schwangere selbst als psychophysiologische Umwelt des Ungeborenen konfiguriert wurde, die sich ihrerseits wiederum in Kontakt einer weiteren Umwelt befindet (Arni 2018: 171).

In diesem Zuge hatten sich in medizinischen Konzepten weiblicher Körperprozesse zwei Stadien etabliert: die Menstruation und die Ovulation. Ende des 19. Jahrhunderts postulierte der britischen Arzt James Hobson Aveling (1874) in einem gynäkologischen Fachaufsatz noch ein drittes Stadium: die Nidation – die Einnistung des befruchteten Eis im Uterus. Schon vorher hatten medizinische Konzepte – wie das von Karl Ernst von Baer (1827) entwickelte – ein „Ei des Säugetiers“ beschrieben (Vienne 2015). In der Wahl des Terminus ‚Ei‘ für das identifizierte Objekt im weiblichen Körperinneren figurierte die Vogelwelt als analoges Modell zur Menschenwelt – und tatsächlich zog von Baer in seinem Text immer wieder Vergleiche zwischen den Eiern von Vögeln und anderen eierlegenden Tieren wie Fröschen. Auch Aveling (1874) berührte in seinem Aufsatz zur Nidation die tierischen Assoziationsräume rund um das ‚Ei‘. Denn in seinem Artikel verglich er explizit den Aufbau der Uterusschleimhaut mit dem Nest, das Vögel für ihre Eier bauen würden. Avelings Artikel wurde früh rezipiert, so findet sich etwa 1875 in den *London Medical Records* eine Zusammenfassung seines Nidationskonzepts. Einige Jahrzehnte später, mit dem Aufstieg der Endokrinologie und dem hormonellen Phasenmodell der Schwangerschaft, verbreitete sich die Begriffe der Nidation bzw. Einnistung endgültig. Ausdrücke etwa zur „Einnistung des Eis in den Fruchthalter“ tauchten nun auch in medizinischen Lehrbüchern und in einigen populärwissenschaftlichen Texten auf (z. B. Poll ca. 1913: 21). Auf diese Weise wurde der Uterus mit der aufgebauten Gebärmutterschleimhaut nicht nur in medizinischen Konzepten zum ‚Nest‘ für den Embryo. Sondern auch in weiter verbreiteten Wissensbeständen zirkulierte die Idee des weiblichen Körpernests.

Insgesamt steht die beginnende Formation der Vorstellung eines Nestbauinstinktes schwangerer Frauen in dieser Periode also mit einer zweifachen Bedeutungsverschiebung des Nest-Begriffs in Verbindung: dem Nest als Metapher für den liebevoll gepflegten und hygienisch gestalteten Wohnraum der Familie und, mit dieser metaphorischen Verschiebung sicherlich zusammenhängend, dem Nest als Uterus. Auf diese Weise erschien die Schwangere doppelt als Mutter und als Hausfrau – die ihr inneres wie äußeres Nest, von Natur sauber und einladend hielt. Entsprechend erwähnte Helene Deutsch beide Konnotationen des Nestes. Sie sprach vom Familiennest ebenso wie davon, dass endokrine Prozesse dem „Ei ein Nest im mütterlichen Körper“ bauen würden (Deutsch 1954: 133).

Als weitere Vorbedingung hatte sich auch parallel die Vorstellung der Psyche in der Schwangerschaft erweitert. Anders als noch im 19. Jahrhundert fanden ab den ersten Jahrzehnten des 20. Jahrhunderts zunehmend auch mütterliche Regungen Eingang. In einem Ratgeber war nun zum Beispiel an einer Stelle vom „Instinkt der Mutterschaft“ zu lesen (Meyer-Rüegg 1915: 26), ebenso erwähnten einige medizinische Studien zur Schwangerschaft gelegentlich ein „Mutterschaftsgefühl“ (Siegel 1919: 202). Parallel dazu begannen auch Psychologisierungsprozesse von Mutterliebe (Schütze 1986; Vinken 2001). Dabei wandten sich gerade ab den 1920er Jahren einige psychoanalytisch geprägte Ratgeber direkt an eine weibliche Leserschaft, um ihr das Glück der Mutterschaft nahe zu bringen, das der vermeintlich natürlichen Disposition aller Frauen entsprechen sollte. Vor diesem Hintergrund nahmen Beschreibungen von Muttergefühlen immer mehr zu – spätestens ab den 1950er Jahren sollte sich die Schwangere bereits als liebende und zufriedene Mutter fühlen. An diese Mütterlichkeit fand ein Konzept wie das Nestbauen, das nun bereits als mütterliches Verhalten definiert war, leichter Anschluss.

## Das zirkulierende Nest – Der Nestbauinstinkt ab den 1970er Jahren

Bis in die 1950er Jahre wurde der Nestbautrieb in der menschlichen Schwangerschaft nur in relativ wenigen akademischen Publikationen erwähnt. Es sollte noch zwei bis drei Jahrzehnte dauern, bis das Wissen zum Nestbauinstinkt im Feld der Ratgeberliteratur ankam. Dort breitete sich das Konzept massiv aus, bis es heute in fast jedem Schwangerschaftsratgeber benannt wird. Der Aufstieg diesbezüglichen Wissens begann ab den frühen 1970er Jahren in einigen englischsprachigen Publikationen: Beispielsweise war im Geburtsratgeber der US-amerikanischen Ärztin Mary Jane Hungerford vom „nesting instinct“ in Bezug auf das Einrichten des Kinderzimmers zu lesen (Hungerford 1972: 26). Auch ein Buch, das Schwangeren Gymnastik für eine ‚natürliche‘ Geburt nahelegte, verwendete den Begriff „nesting instinct“, wobei es Analogien zwischen weiblichen und tierischen Nestbauverhaltens herstellte (Hartman 1975: 4).

In der deutschsprachigen Ratgeberliteratur fand der Begriff einige Jahre später Verwendung. Zunächst wurden hier konzeptnahe Vorstellungen erwähnt: Einige Bücher berichteten von „Tätigkeitsdrang (großes Reinemachen, Wäsche, Vorratseinkäufe, Besuche!)“ (Liechti-von Brasch & Bretscher 1973: 168) und „Arbeitswut“ (Weilandt 1983: Klappentext). Dann, spätestens ab den 1980er Jahren, wurde auch hier wortwörtlich von einem „Nestbauinstinkt“ gesprochen, zu dem vor allem Putzen, Einkaufen und Einrichten zählten (Stoppard 1986: 171). Die Zeitverzögerung im deutschsprachigen Raum um ungefähr ein Jahrzehnt lässt vermuten, dass es sich bei der Verbreitung des Begriffs vom ‚Nestbauen‘ teilweise um eine Art (Re‑)Import aus der englischen Sprache handeln könnte. Denn in dieser Phase ist allgemein eine zunehmende ‚Anglophonisierung‘ der deutschsprachigen Schwangerschaftsratgeber festzustellen, wurden nun doch einige auflagenstarke englischsprachige Bücher wie das der britischen Ärztin Miriam Stoppard für den deutschen Markt übersetzt. Dieser leichten zeitlichen Verschiebungen zum Trotz – ab den 1980er Jahren schien die Vorstellung des Nestbautriebs in populären Darstellungen von Schwangerschaft fest verankert.

Insgesamt ist das in die Alltagskultur übertragene Konzept des Nestbauinstinkts von drei Aspekten geprägt, in denen sich die Tendenzen einiger vorangehender Wissensmuster verfestigten: Erstens ging es hier nun um das Instinktverhalten von Frauen, das aber oft mit dem von Tieren verglichen wurde. Dabei standen neben dem weiterhin prominenten Nestbauinstinkt von Vögeln vor allem Säugetiere im Vordergrund. Zweitens fungierten nun Hormone uneingeschränkt als körperliches Erklärungsmodell. Wurden diese spezifiziert, so ging es um ‚weibliche‘ Geschlechtshormone bzw. ‚Schwangerschaftshormone‘, insbesondere um Progesteron. Und drittens war die geschlechtliche Codierung des Instinkts in diesem Wissensfeld eindeutig feminin, es handelte sich um ein Verhaltensrepertoire, das Menschenfrauen und Tierweibchen charakterisierte.

Sucht man nach den Faktoren, weshalb sich das Wissen zum Nestbauinstinkt genau in diesem Zeitraum und in dieser spezifischen Sphäre der Ratgeberliteratur verbreitete, so ist hier von einem Komplex miteinander interagierender Praktiken und Diskurse auszugehen. Die Verbindung dieser heterogenen Bereiche wurde, so möchte ich argumentieren, dadurch erleichtert, dass das Nestbauen in dieser Zeit schon längst nicht mehr nur ‚epistemisches Ding‘ war, sondern es sich längst auch zu einem ‚metaphorischen Ding‘ transformiert hatte. Jürgen Link betont, dass Metaphern eine „Übertragbarkeit“ von Vorstellungen in „andere Praxis- und Diskursbereiche“ ermöglichen (1983: 13). Und Christina Brandt (2004) zeigte in ihrer Fallstudie zur Virusforschung, dass Metaphern als Transmitter zwischen wissenschaftlichen und nicht-wissenschaftlichen Bereichen fungieren, wobei sie durch ihre Anschaulichkeit und relative Offenheit vor allem in populären und semipopulären Texten zirkulieren. Welche Bereiche wurden also durch die Metapher des Nests miteinander verbunden und begünstigten ihrerseits die Zirkulation des Nestbauinstinktes?

Einen Bereich bilden Diskurse zu Schwangerschaft, in denen in den 1970er und 1980er Jahren eine Psychologisierung und Emotionalisierung erfolgte, die für die Verbreitung des Nestbautriebes von Bedeutung war (Malich 2017). Hierbei spielten zeitgenössische Konzepte von Mütterlichkeit eine Rolle, in denen – wie Susanne Schmidt im vorliegenden *Special Issue* beschreibt – die Mutter konzeptuell zur Umwelt des Kindes wurde. In diesem Zusammenhang kam der in dieser Zeitperiode auftauchenden *attachment theory* bzw. Bindungstheorie große Bedeutung zu. Die Grundlagen der Theorie veröffentlichte der britische Psychologe John Bowlby 1958, in den 1960er und 1970er Jahren erlangte sie zunehmend an Einfluss (Crouch & Manderson 1995; Vicedo 2013). Bowlby postulierte, dass Kinder ein instinktives Bedürfnis hätten, sich an Bezugspersonen (hier gedacht als die Mutter) zu binden und diese zu lieben – ebenso wie Mütter einen natürlichen Instinkt besäßen, ihren Nachwuchs zu lieben und umsorgen.

Neben psychoanalytischen Theorien und eigenen Erfahrungen in der Kindertherapie war für Bowlbys Argumentation der Vergleich mit der Tierwelt grundlegend. Dieser erfolgte durch den Rekurs auf die Arbeiten eines Wissenschaftlers: Konrad Lorenz (Vicedo 2013). Seine Idee der Verhaltensprägung von Graugänsen war ein essenzieller Bestandteil von Bowlbys Theorie des Bindungsbedürfnisses von Kindern. Die Bindungstheorie legitimierte die Übertragung von Lorenz’ Ethologie auf die menschliche Psyche nun endgültig – und trug noch zusätzlich zu seiner Bekanntheit im deutsch- und englischsprachigen Raum bei. Denn Lorenz’ Arbeiten genossen nach dem Zweiten Weltkrieg sowieso schon hohen Bekanntheitsgrad – durch ethologisch informierte Fernsehsendungen wie *Rendezvous mit Tier und Mensch*, durch den Nobelpreis, der 1973 an Lorenz und seinen Kollegen Nikolaas Tinbergen verliehen wurde, und vor allem durch Lorenz’ populäre Tierbücher (Taschwer 2002): 1949 erschien sein Besteller *Er redete mit dem Vieh, den Vögeln und den Fischen*, 1978 wurde das *Jahr der Graugans* veröffentlicht. In den Büchern anthropomorphisierte Lorenz seine Gänse in ähnlicher Form wie es bereits die Forscher des 19. Jahrhunderts getan hatten. In letzterer Publikation erklärte er etwa, „daß die Graugans in vielen entscheidenden Punkten ein dem Menschen analoges Familienleben hat“ und beschrieb entsprechend „die Eheschließung bei Gänsen“ und die „gemeinsame Liebe zu den Kindern“ (Lorenz 1982: 17; 20). Bei der Beschreibung des Gänselebens widmete er dem Nestbau viel Aufmerksamkeit und nahm zudem mitunter explizit Bezug auf Bowlbys psychologische Ansätze (ebd: 31). Der aus Lorenz’ Arbeiten bekannte Nestbauinstinkt mag so auch durch die Bindungstheorie Eingang in Diskurse zu Schwangerschaft gefunden haben.

Einen weiteren Bereich bildete die reformorientierte Geburtshilfe. Hier dienten die Bindungstheorie und Verweise auf eine idealisierte weibliche ‚Natur‘ dazu, zeitgenössische Medizinkritik und alternative Praktiken zu unterstützen, deren historische Entwicklung unter anderem Anne Harrington (2009) analysiert hat. So fungierte der Verweis auf die mütterlich-kindliche Bindung in Geburtsstationen als wirkungsvolles Argument gegen die in westlichen Ländern der Nachkriegszeit verbreitete medizinische Praxis, Neugeborene von ihren Müttern räumlich zu trennen (Crouch & Manderson 1995). Auf diese Weise setzte sich ab den 1970er Jahren in Kliniken die gemeinsame Unterbringung von Mutter und Baby in einem Raum – das sogenannte Rooming-in – durch. Die ethologisch geprägte Bindungstheorie überschnitt sich auch mit der natürlichen Geburtsbewegung der 1970er und 1980er Jahre, die noch stärker von alternativer Heilkunde geprägt war. Vor diesem Hintergrund ist es wohl wenig erstaunlich, dass der ethologisch geprägte Begriff des ‚Nestbautriebes‘ relativ früh gerade in denjenigen populären Schriften zu Schwangerschaft erschien, die die ‚naturwissenschaftlich orientierte Medizin‘ kritisierten und vermeintlich natürliche Geburtspraktiken und emotional-therapeutische Selbsterfahrung vertraten: von *Exercises for True Natural Childbirth* (Hartman 1975) über *Yoga for Pregnancy and Birth* (Shandler & Shandler 1979) bis hin zum deutschsprachigen und ebenfalls naturheilkundlichen Ratgeber *Gesunde Schwangerschaft – glückliche Geburt* (Liechti-von Brasch & Bretscher 1973). Das Zusammenwirken von psychologischer Bindungstheorie, den Vogelgeschichten der Ethologie und alternativmedizinische Geburtspraktiken scheint so als weiterer Katalysator für die Verbreitung des Nestbautriebes.

## Ein Nest kaufen, ein Nest putzen

Inhaltlich sind zwei Facetten besonders charakteristisch für das Wissen zum menschlichen Nestbauinstinkt, das sich ab den 1970er Jahren verbreitete, nämlich das Konsumieren und das Putzen. Beide Aspekte mögen ebenfalls Aufschluss über die kontextuellen Möglichkeitsbedingungen dieses Wissens geben. Als erste Facette trat im Wissen zum Nestbauinstinkt nun eine Dimension der Nest-Metapher noch deutlicher hervor, die ihr schon inhärent gewesen war: die Zwischenposition des Nests als sowohl natürliche wie auch artifizielle Umwelt. Denn die nestbauende Schwangere schien in den zeitgenössischen Beschreibungen trotz aller tierweltlichen Analogien überraschend wenig am ‚naturnahen‘ Nestbau von Gänsen oder Hasen interessiert – vielmehr richtete sich das weibliche Verhalten nun stark an der Konsumkultur westlicher Gesellschaften aus.

Liest man die zeitgenössischen Ratgeber, so äußert sich der Trieb, neben dem Putzen, vor allem im Kaufen von Produkten, dem Erwerb einer Babyausstattung, dem Renovieren und Dekorieren des Hauses sowie dem Einrichten und Ausstaffieren eines Kinderzimmers. Schon bei Deutsch (1954) spielte ja das „perfekt eingerichtete Kinderzimmer“ und die „Babyausstattung“ beim Nestbauen eine große Rolle. Später erhielten Konsumpraktiken in vielen Ratgebern einen festen Platz. In frühen deutschen Schriften war zunächst noch relativ bescheiden von „Vorratseinkäufen“ zu lesen (Liechti-von Brasch & Bretscher 1973: 168). Zu einer englischsprachigen Darstellung des „nesting instincts“ zählten schon folgende Tätigkeiten: „readying the home, decorating a room, or buying a crib and layette“ (Simons & Pardes 1977). Ähnlich gehörte es im Ratgeber Stoppards zum Nestbautrieb, „zu überprüfen, ob alles für die Ankunft eines Babys fertig ist – das Zimmer, die Kleidung, die Ausrüstung“ und gegebenenfalls fehlende Gegenstände einzukaufen (Stoppard 1986: 165). Rund zwei Jahrzehnte später erfahren auch werdende Väter in einem englischsprachigen Ratgeber davon: „You see your partner transform as the baby grows, and she may start planning what to buy (not more clothes!) and how to decorate the baby’s room. This is called ‚the nesting instinct‘ and you pretty much have to go with it“ (Korn et al. 2012: Chapter 4, Publikation ohne Seitenzahlen). Solchen Darstellungen gemäß schien sich der Nestbauinstinkt also primär in einem Kaufinstinkt auszudrücken und die Nestumwelt war ein Konsumprodukt.

Darin spiegeln sich die verbesserten ökonomischen Bedingungen und aufsteigende Konsumkultur, welche die USA in der Nachkriegszeit prägten und in den meisten westlich-europäischen Ländern spätestens in den 1970er und 1980er Jahren ebenfalls an Einfluss gewannen (Sassatelli 2007). Ab der zweiten Hälfte des 20. Jahrhunderts stieg, im Gefolge von Massenproduktion und Wirtschaftsaufschwung, der generelle Lebensstandard: Wohnungseinrichtung, Dekoration und Kleidung waren erschwinglicher. Sie waren nicht mehr Anschaffungen fürs Leben, sondern sie konnten relativ kurzentschlossen gekauft und, je nach Mode, schnell wieder ausgetauscht werden.

Zudem entwickelte sich Kindheit verstärkt zu einer ökonomischen Kategorie mit einer ausdifferenzierten Produktpalette und breitem medialen Marketing (Cook 2013; Cross 1997). Vor dem Hintergrund einer wachsenden Konsumkultur wurden Mütter zur bevorzugten Zielgruppe für Kinder- und Gesundheitsprodukte. Dies betraf schon die Schwangerschaft: Es galt im größer werdenden Markt Babyprodukte zu wählen, sei es Strampler, Kuscheltiere, Kinderbettchen, Windeln, Spieluhren oder Nahrungsergänzung. Diese veränderten Konsumpraktiken lassen sich auch in den Ratgebern beobachten, in denen ab den 1970er Jahren immer differenziertere Produkte vorgestellt, immer durchdachtere Vorbereitungsmöglichkeiten beschrieben werden. Die spezifische Formation des Wissens zum Nestbauinstinkt ab den 1970er Jahren ist also auch Folge von ökonomischen Dynamiken. Sie ergibt sich daraus, dass die heimische Umwelt des Nests detaillierter ausstaffiert werden konnte.

Die zweite prominente inhaltliche Dimension des menschlichen Nestbauinstinkts bildet die Hausarbeit: das Putzen, Waschen und Aufräumen, also klar weiblich konnotierte Arbeiten (von Bose 2017). Für Arianne Shahvisi (2020) ist in ihrer Analyse diese Dimension sogar so wichtig, dass sie als Grund für den Aufstieg des Nestbauinstinkts eine Vergeschlechtlichung von Hausarbeit annimmt. Und tatsächlich ist Putzen in den Quellen omnipräsent. So sprach Döring bereits von „Putzaktionen“ (1958b: 150), ebenso war in den eingangs erwähnten kontemporären Zitaten von einem „Putzfimmel“ die Rede (Grünebaum & Okko 2010: 279). Auch englischsprachige Bücher definieren als zentrales Merkmal des Nestbauinstinkts „a push to clean, organize and get everything ready for when baby comes“ (Lluch et al. 2008: 183). Dabei kommt es durchaus zu Überschneidungen zwischen den Metaphern des Körpernests und des zu putzenden Wohnungsnests. So ist in dem weit verbreiteten Ratgeber einer naturheilkundlich orientierten Hebamme zu lesen:

Es ist sicherlich viel Wahres daran, dass Putzen und Vorbereiten auf die Geburt viel miteinander zu tun haben. […] In der Schwangerschaft beginnt einige Wochen früher, was im weiblichen Zyklus normalerweise einige Tage vorher stattfindet. Sinnbildlich ist zwischen Bluten, Gebären und Putzen viel Ähnlichkeit: Nämlich Abbruchblutung und frischer Schleimhautaufbau der Gebärmutter. Wieso dann nicht auch Putzen und Reinemachen der Wohnung? (Stadelmann 1999: 143)

Hier fungiert die Schleimhaut der Gebärmutter als direktes Analogon zum Wohnungsinneren, das Gebären fungiert als Analogon zum Putzen – so dass eine unmittelbare Biologisierung weiblicher Reinigungsarbeit auf metaphorischer Ebene stattfindet. Woher kommt der Fokus auf das Putzen, der in den Darstellungen des tierischen Nestbauinstinkts fehlte?

Das Wissen zum instinktiven Putzen in der Schwangerschaft stieg parallel mit der zweiten Welle der Frauenbewegung der 1970er Jahre auf. Die Emanzipationsbewegung stellte die hegemoniale Geschlechterordnung in Frage, kritisierte die Beschränkungen der weiblichen Rolle auf Hausfrau und Mutter. Gerade gebildete Frauen lehnten Putzen und Versorgen – die Herstellung eines gemütlichen und liebevollen Nests für die Familie – als „Arbeit aus Liebe“ (Duden & Bock 1977) zunehmend ab. Allerdings änderten sich hegemoniale weibliche Rollenzuschreibungen angesichts solch drohender Auflösungserscheinungen nicht unbedingt – sie wurden vielmehr anders begründet. So beschreibt Rebecca Jo Plant (2010) in ihrer Geschichte von Mutterschaft, dass in dieser Zeit die ältere Idee der ‚moralischen Mutterschaft‘, die Mühe und Pflicht betonte, zwar suspendiert wurde, nun aber Mutterliebe in einen psycho-biologischen Trieb transformiert wurde. Mütterlichkeit erschien nun weniger als Arbeit und mehr als natürliches Bedürfnis. Dadurch erfuhr die traditionelle Frauenrolle zugleich eine gewisse Entwertung und zugleich eine neue Legitimation über naturwissenschaftlich geprägte Begründungsmuster (Vicedo 2013).

In Bezug auf den Nestbautrieb ist von ähnlichen Mechanismen auszugehen. Schließlich handelt es bei den instinkthaften Verhaltensweisen um traditionelle weibliche Tätigkeitsmuster, die zum Komplex von Mütterlichkeit zählen. Speziell in Bezug auf Reinigungsarbeiten intensivierten sich zudem Erwartungen in dieser Zeitperiode noch. Denn in den 1960er und 1970er Jahren stiegen, parallel zur Verbreitung von elektrischen Geräten wie Staubsauger und Waschmaschine, die Sauberkeitsmaßstäbe (Schwartz Cowan 1985). Die ‚gute Hausfrau‘ musste die Zeitersparnis durch die Technologien nutzen, um ihre Reinlichkeit durch ein strahlend saubereres Nest unter Beweis zu stellen. Als hormonell begründeter Trieb wirkte das Putzen im Kontext des Nestbauens in der Schwangerschaft nun aber nicht als Ausrichtung an einer gesellschaftlich-technologisch geschaffenen Norm, sondern als unveränderliche biologische Konstante. Damit erfolgte eine Essentialisierung von Rollenmustern, die mit dazu beitrug, eine umstrittene Geschlechterordnung zu stabilisieren.

Die Biologisierung weiblich konnotierter Arbeiten durch den Nestbauinstinkt ging – wie es auch Vicedo (2013) beschreibt – teilweise mit deren Entwertung einher: Hausarbeit war nun nicht weniger moralische Pflicht, deren Erfüllung Ausdruck von Willensstärke war, sondern hormonell determinierter Drang. Dies schlägt sich in den Quellen dadurch nieder, dass Beschreibungen des Nestbauinstinkts oft ironisiert werden oder gar als riskant, latent pathologisch oder zumindest kontrollbedürftig erscheinen. Beispielsweise erklärt ein Ratgeber seiner schwangeren Leserin, der „Nestbautrieb“ fühle sich an „wie ein heftiger Koffein- und Adrenalinstoß, gemischt mit Euphorie, Panik und einer leichten Zwangsneurose“ (Fraser 2012: 98). In dem Buch folgt eine Liste an Vorsichtsmaßnahmen, die die Schwangere beachten solle, wenn sie ihrem Nestbauinstinkt nachgebe. Dazu gehört etwa, nicht mehr auf Leitern zu steigen (Sturzgefahr), Materialien mit chemischen Dämpfen zu vermeiden in seinem ästhetischen Urteil bei Einrichtungsfragen zu misstrauen und stattdessen mehr auf „Ratschläge Ihres nicht schwangeren und daher in seinem Urteilsvermögen nicht ganz so eingeschränkten Partners“ zu hören (Fraser 2012: 99). Auf diese Weise wurde mit dem Wissen zum Nestbauinstinkt eine schwangere Subjektivität konstruiert, deren Rationalität und Autonomie durch ihre ‚natürlichen‘ Triebe eingeschränkt sind und die deswegen auf Rat und Kontrollmaßnahmen angewiesen scheint. Dies korrespondiert mit Vorstellungen von Schwangerschaft als Risiko, die ab den 1980er Jahren zunahmen und die das schwangere Subjekt zugunsten präventiver Regulierung außer Kraft setzten (Kneuper 2004; Kukla 2005; Sänger et al. 2013).

Zugleich illustriert der schmale Grat zwischen natürlichem Instinkt und dessen gleichzeitiger Überwachung auch die Ambivalenz, die in der Denkfigur des ‚Nestes‘ als spezifische natural-anthropogene Umwelt steckt. Das Nest als Raum der Familie, der diese wie eine Hülle vor den Einflüssen des Außen schützt, bot gerade wegen seines Doppelcharakters als ‚natürliche‘ und ‚gemachte‘ Umwelt einen wichtigen Angriffspunkt für medizinisches, pädagogisches und psychologisches Wissen sowie für biopolitische Regulationsmaßnahmen. Wenngleich die ‚Instinkte‘ und ‚das Nest‘ gemeinhin dem Bereich der Natur zugeordnet werden, so wäre es also zu wenig, im Nestbautrieb lediglich eine Naturalisierung weiblicher Haus- und Sorgearbeit zu sehen. Vielmehr wurde diese Arbeit so zum natural-kulturellem Phänomen, das sowohl Teil der Natur ist als auch der sozialen Regulation bedarf.

## Schlussbemerkungen: Die Frau als Nest, das Nest als Umwelt

In diesem Aufsatz wollte ich drei Fragenkomplexe beantworten, nämlich auf welche Weise sich das Wissen um einen Nestbautrieb in der Schwangerschaft, wie hierbei das Nest als spezifische natural-anthropogene Umwelt hergestellt wurde und in was für Wechselwirkungen Vorstellungen von Geschlecht und Umwelt traten.

Zusammenfassend kann die Zirkulationsgeschichte des Nestbautriebes in zwei Phasen der Vergeschlechtlichung unterteilt werden. In der ersten Phase dominierte eine neutrale bis maskuline Konnotation. Hier formierte sich das Wissen zum Nestbauinstinkt in den Feldern der bürgerlichen Naturbeobachtung, der Zoologie und Verhaltensbiologie im 19. und frühen 20. Jahrhundert. Das Nestbauverhalten meist männlicher Vögel bildete ein zentrales epistemisches Objekt, wobei das Nest in manchen Analogien zur Menschenwelt als ganzes Haus oder Wohnung figurierte. In der zweiten Phase begann eine partielle Feminisierung des Konzepts, die ab den 1930er Jahren einsetzte, als es in den Feldern der Ethologie und der endokrinologischen Tierforschung zum epistemischen Objekt wurde. Diese führten das Nestbauen auf hormonelle Auslöser zurück und definierten es als Eigenschaft von Tierweibchen. Kurz darauf überkreuzten sich die Diskursstränge vom tierischen Nestbauinstinkt und menschlicher Schwangerschaft. So fand das Konzept ab den 1940er Jahren zunächst vereinzelt Eingang in psychoanalytische und psycho-endokrinologische Fachliteratur zur weiblichen Psyche. Dazu trug bei, dass das ‚Nest‘ zur absoluten Metapher mit doppelter Konnotation geworden war: Das Nest stand für die Umwelt des Kindes im Körperinneren, die Gebärmutter; und es stand für den Innenbereich des Hauses, für die heimische Umwelt, die es zu gestalten galt. Schließlich wanderte der Nestbauinstinkt in den 1970er und 1980er Jahren in das Genre der Ratgeberliteratur und verbreitete sich, so dass das Wissen heute fest zu den psychischen Charakteristika der Schwangerschaft zählt. Inhaltlich stehen dabei nun Tätigkeiten des Putzens, Einrichtens und Konsumierens im Vordergrund. Diese deuten auf konstitutive Kontextbedingungen in der zweiten Hälfte des 20. Jahrhunderts hin, die sich um die Metapher des Nests gruppieren: Den Aufstieg der Schwangerschaft zur Konsumkategorie und die Hinterfragung vermeintlich weiblicher ‚Pflicht‘ zur Hausarbeit, die teilweise in der Stabilisierung dieser Arbeitsbereiche durch ihre Biologisierung mündete.

Als Denkfigur waren im Nest als Umwelt bestimmte Paradoxa angelegt. Denn das Nest der Tiere wurde zwar dem Bereich der Natur zugeteilt, war aber hier schon bereits Teil einer hergestellten Natur – als ‚innere Umwelt‘ sollte das Nest gegen eine natürliche Außenwelt, gegen Wetter und Fressfeinde, schützen. In seiner anthropomorphisierten Form war die Metapher des Nestes zudem von Kulturpraktiken, sozialen Normen und geschlechtlichen Zuschreibungen geprägt. Im Rahmen seiner graduellen Feminisierung wanderte das ‚Nest‘ zunehmend nach innen. Figurierte das ‚Nest‘ des Vogelmännchens metaphorisch noch eher für das Haus als Gebäude, so verengte sich das Nest der schwangeren Frau auf die Inneneinrichtung bzw. ihr Körperinneres. Dabei war das Nest als Ort der Versorgung zunehmend auf die kindliche Entwicklung zentriert. Das Konzept des ‚Nestbauinstinkts‘ legitimierte nicht nur die Vergeschlechtlichung von damit einhergehenden Tätigkeiten – sondern es machte diese Arbeiten dergestalt zum natural-kulturellem Phänomen, das sowohl Teil der Natur ist, als auch der sozialen Regulation, Intervention und Beratung bedarf.

Das Wissensobjekt ‚Nestbauinstinkt‘ unterscheidet sich von anderen Objekten, zu denen eine historische Epistemologie geschrieben wurde, beispielsweise der Proteinsynthese (Rheinberger 2006) oder dem Tabakmosaikvirus (Brandt 2004). Es entstand nicht primär im Labor, wenngleich Experimentalsysteme im Labor durchaus eine Station seines Werdegangs bildeten. Noch stärker als bei anderen Objekten handelte es sich um ein zirkulierendes Konzept, das sich in nicht ausschließlich, aber vor allem in populären Wissenskulturen formierte und modifizierte. Für die Formation des Nestbauinstinkts spielten die von spezifischen akademisch-epistemischen Systemen vorgegebenen Bedingungen eine Rolle – etwa die der zoologischen Beobachtungswissenschaften oder der endokrinologischen Experimentalsysteme. Allerdings war für die Wissensgeschichte des Nestbauinstinkts die sprachliche Metapher von größerer Bedeutung als für viele Gegenstände, die in erster Linie wissenschaftshistorisch zu behandeln sind. Denn als ‚metaphorisches Ding‘ fungierte das Nestbauen als wichtiger Umschlagplatz für kulturelle Normen, Vorstellungen geschlechtlicher Arbeitsteilung und deren wandelnde historische Begründungen. Zugleich fungierte die Metapher als Bindeglied zwischen heterogenen Praktiken und Diskursen. Daraus ergibt sich ein weiteres Paradoxon des ‚Nests‘, diesmal bezüglich seiner gesellschaftlich-kulturellen Funktion: Zwar bezeichnet der Begriff selbst die Vorstellung eines Innenraums, eines begrenzten Bereiches – als Metapher führte der Begriff aber gerade zur Verbreitung und Zirkulation dieser Vorstellung. Und so leben wir heute in einer Gesellschaft, in der Nester gebaut werden: ‚Nido‘ (italienisch für ‚Nest‘) ist der Titel einer bekannten Familienzeitschrift, ‚Babynest‘ ist der Produktname für Säuglingsbetten und ‚Google Nest‘ verkauft das digital gesteuerte Wohnen. Verantwortlich für diese Nester sind fast immer die Frauen.

## Footnotes

1 Im Jahr 2020 verzeichnete das Portal Google Scholar nur insgesamt elf Zitationen für die sieben Jahre seit der Publikation des Aufsatzes, was in den Lebenswissenschaften einer kaum vorhandenen Rezeption entspricht und die Marginalität dieses Themas in der aktuellen empirischen Forschung illustriert.

2 Genannt seien hier nur einige der zahlreichen historischen Analysen, wie etwa Arni (2018); Duden, Schlumbohm & Veit (2002); Evans & Meehan (2017); Hanson (2004); Hopwood (2015); Hornuff (2014); Labouvie (2000); Malich (2017); Nemec & Zimmer (2019); oder eher sozial- und kulturwissenschaftlich ausgerichtete Werke: beispielsweise Hirschauer et al. (2014); Müller & Zillien (2016); Schadler (2013); Seehaus (2015); Seigel (2014); Villa, Moebius & Thiessen (2011).

3 Gesucht wurden zwischen Juli 2018 und Dezember 2019 die Begriffe ‚Nest & Schwangerschaft‘, ‚Nestbauinstinkt‘, ‚Nestbautrieb‘, ‚Nesting‘, ‚Nesting Instinct‘, ‚Nidation‘ und ‚Nest‘ in Medline, Pubmed, Google Books, Google Scholar und Ngram. Anschließend wurden angezeigte Publikationen recherchiert und analysiert.

999 Olszynko-Gryn, Jesse 2012. *Locating the Pregnancy Hormone, 1928–1945. The Aschheim-Zondek test and the Controversy over the Hormonal Placenta. *Unpublished paper. Cambridge: Cambridge University, Department of History and Philosophy of Science.
